# The Different Brain Mechanisms of Object and Spatial Working Memory: Voxel-Based Morphometry and Resting-State Functional Connectivity

**DOI:** 10.3389/fnhum.2019.00248

**Published:** 2019-07-19

**Authors:** Zhiting Ren, Yao Zhang, Hong He, Qiuyang Feng, Taiyong Bi, Jiang Qiu

**Affiliations:** ^1^Key Laboratory of Cognition and Personality (SWU), Ministry of Education, Chongqing, China; ^2^School of Psychology, Southwest University, Chongqing, China; ^3^Center for Mental Health Research in School of Management, Zunyi Medical University, Zunyi, China

**Keywords:** visual working memory, structure, resting-state functional connectivity, object working memory, spatial working memory

## Abstract

In working memory (WM), the ability to concurrently integrate different types of information and to maintain or manipulate them promotes the flow of ongoing tasks. WM is a key component of normal human cognition. In this study, we applied a combined voxel-based morphometry (VBM) and resting-state functional connectivity (rsFC) analysis to investigate the relationship between the ability of object and spatial working memory (WM), and regional gray matter density (GMD), as well as intrinsic functional connectivity. The VBM analysis showed a positive correlation between the individual difference of object WM and GMD in the right middle occipital gyrus (MOG) and the left superior temporal gyrus (STG), which are responsible for coding object information and processing the shape of an object. The individual difference of the spatial WM was positively related to GMD in the right middle frontal gyrus (MFG) located in the dorsolateral prefrontal cortex (dlPFC), which confirmed that it is an important region for memory stores and maintains WM spatial representations. Further functional connectivity analysis revealed that the individual difference of object WM was significantly correlated with the rsFC of right intraparietal sulcus (IPS) – left postcentral gyrus (PostCG)/right precentral gyrus (PreCG)/left Supplementary Motor Area (SMA). While the capacity of spatial WM was significantly associated with the FC strength of the left dlPFC – left precuneus, right dlPFC – right MFG, and the left superior frontal sulcus (SFS) – left SMA/ right inferior parietal lobe (IPL). Our findings suggest that object WM is associated with the structure and functional organization of the brain regions involved in the ventral pathway (occipital – temporal regions) and the capacity of spatial WM is related to the dorsal pathway (frontal – parietal regions).

## Introduction

Working memory (WM) refers to a limited system that provides for the temporary storage and manipulation of information. It is a basic mechanism for many highly complex cognitive activities. Thus, understanding the neural mechanisms of WM is crucial to the further study of high-level cognition. WM consists of four subcomponents: a central executive system for attentional control, a phonological loop for the storage and manipulation of verbal materials, a visual-spatial sketchpad for object and spatial information, and an episodic buffer for storage of information (Baddeley, 2003). The visual-spatial sketchpad is called “visual working memory” (VWM) and has two subsystems: one for object WM processing object information and one for spatial WM processing spatial information.

Researchers have done a lot of work to explore the neural mechanism of object and spatial WM. The dorsal occipitoparietal pathway processes spatial information such as movement, location, and the spatial relationship among objects. The posterior parietal cortex (PPC) plays an important role in spatial WM (Alekseichuk et al., 2017). In contrast, the ventral occipitotemporal pathway is essential for processing object information such as patterns and color (Felleman and Van Essen, 1991). The medial temporal lobe (MTL) is significantly active during object WM tasks (Picchioni et al., 2007), and lesions in the MTL impair the ability to discriminate between similar objects (Knutson et al., 2012) and impair the face WM (Ezzyat and Olson, 2008).

Visual working memory also relies heavily on the frontal lobe. A temporal-frontal circuit is considered to be related to pattern recognition and a parietal-frontal circuit is considered to be related to spatial information (Wager and Smith, 2003). Many imaging studies have shown that the ventrolateral prefrontal cortex is consistently activated during WM for object information (Prabhakaran et al., 2000; Mohr et al., 2006b), however, evidence from neuroimaging studies indicated that the dorsolateral prefrontal cortex (dlPFC) (Yin et al., 2013; Pearce et al., 2014) was significantly activated during spatial WM tasks, and the superior frontal sulcus has also been proven to be a critical region for spatial WM (Zarahn et al., 1999).

It is worth noting that several imaging studies in humans have found no significant differences in the activation of brain networks for these two types of tasks (Baker et al., 1996; Nystrom et al., 2000), and single cell recording data also showed that many cells across both the dorsal and ventral prefrontal cortex maintain both spatial and object information. Additionally, the results show that lesions of the ventral prefrontal cortex can impair performance in both spatial and object WM tasks (Levy and Goldman-Rakic, 2000).

The reason for these inconsistent results in the prefrontal region regarding functional segregation or integration of WM maintenance is not clear. Most previous VWM-related neuroimaging studies applied functional magnetic resonance imaging (fMRI) and transcranial direct current stimulation (tDCS) to detect the neural mechanism. However, in recent years, more and more studies used both structure and resting-state functional connectivity (rsFC) analyses. Structure imaging allowed us to explore neuroanatomical correlations with differences in human behavior and cognition (Kanai and Rees, 2011) and how the alteration of the structure of the human brain can influence neural function, as revealed in previous studies (Kwaasteniet et al., 2013). Meanwhile, rsFC allowed us to assess the relationship between spontaneous neural activity in different regions of the brain (Fox and Raichle, 2007). The previous study used the Digits-span test to investigate the correlation between the GMV and WM capacity (Tsutsumimoto et al., 2015) and the GMD associated with the performance of Digit background (Richardson et al., 2011). However, no studies focused on the individual difference in both brain structure and functional connectivity associated with object and spatial WM. In the current study, we investigate the brain regions associated with object and spatial information, using structural imaging analyzed by VBM and then extract the obtained region seed regions (ROI) for further rsFC analysis to investigate the associated brain regions of both object and spatial WM.

Based on previous studies (Fox and Raichle, 2007; Kanai and Rees, 2011), we hypothesize that the individual difference in object WM might be related to the brain regions of the temporal and the ventral frontal cortex and the individual difference might be associated with rsFC with the ventral stream. For spatial WM, we hypothesize that the individual difference may be associated with the parietal brain region and the dlPFC and might be related to rsFC in the dorsal stream.

## Materials and Methods

### Participants

Fifty right-handed healthy college students from Southwest University participated in the experiment. Five participants were excluded (four participants for excessive head motion during resting-state fMRI, defined as >3 mm translation in any axis and >3° angular rotation and one participant for poor performance in the WM task), resulting in a final sample of 45 subjects for further analysis (males = 22, females = 23, mean age = 20.11, *SD* = 1.76). All participants had no history of neurological or psychiatric disorders. The participants were recruited on campus and were paid for their participation. After obtaining written informed consent, participants finished the WM task and were subjected to an MRI scan.

### Working Memory Task

We applied the Change Detection Paradigm to test WM (Phillips, 1974), which required subjects to respond to whether two successively presented stimuli were the “same” or “different.” The Change Detection Paradigm was programmed in Matlab R2012a (Math Works Inc.^1^) using the Psychtoolbox software bag.

**FIGURE 1:**
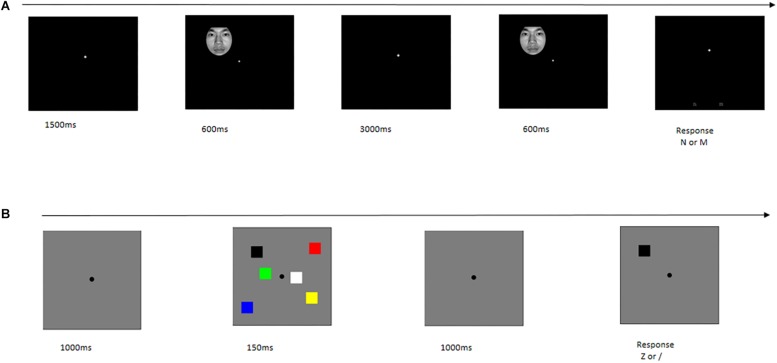
Single trial time-course in behavior task. **(A)** Object working memory. **(B)** Spatial working memory.

Face pictures were used as the major stimuli in object WM. Eight blocks were included, and each block comprised 40 trials. Each trial sequence began with the presentation of a fixation for 1500 ms. Then, a memory item was presented for 600 ms, followed by a 3000 ms blank retention interval. Next, a test item was presented for 600 ms. Subsequently, participants were instructed to press a button. If the test stimulus was the “same” as the memory array stimuli, subjects were instructed to press “N;” otherwise, press “M” (Figure 1A).

Color squares were used as the major stimuli in spatial WM. There were two blocks, and each block was composed of 120 trials. Each trial sequence began with the presentation of a fixation for 1000 ms, followed by the presentation of a memory item for 150 ms. This sequence was followed by a 1000 ms blank retention interval; then, a test item was presented. If the color of the test stimulus was the “same” as the memory array stimulus in this location, participants were instructed to press “Z;” otherwise, press “/” (Figure 1B).

### Data Acquisition

All of the MR images were collected on a Siemens 3T Trio scanner (Siemens Medical, Erlangen, Germany). High-resolution T1-weighted structure images were acquired using a MPRAGE sequence: TR/TE/TI = 1900/2.52/900 ms, FA = 9°, resolution matrix = 256 × 256, slices = 176, thickness = 1.0 mm, and voxel size = 1 mm^3^ × 1 mm^3^ × 1 mm^3^. Resting-state fMRI images were performed by a Gradient-echo Planar Imaging sequence, with scan parameters of TR/TE = 2000 ms/30 ms, FA = 90°, slices = 32, resolution matrix = 64 × 64, FOV = 220 mm × 220 mm, thickness = 3 mm, voxel size = 3.4 mm^3^ × 3.4 mm^3^ × 4 mm^3^. Finally, 242 volumes were acquired for each subject.

### Voxel-Based Morphometry Analysis

The structural MR images were processed with VBM-DARTEL using SPM8 software (Welcome Department of Cognitive Neurology, London, United Kingdom^2^) incorporated in MATLAB 2010a (Math Works Inc., Natick, MA, United States). The images were segmented into gray matter (GM), white matter, and cerebrospinal fluid using the new segmentation tool. Subsequently, we performed registration and normalization by DARTEL in SPM8. The registered images were transformed to MNI space. Finally, the normalized images (GM) were smoothed with a full width at a half-maximum (FWHM) Gaussian kernel of 8 mm. Statistical analyses of GMD data were performed in SPM8. We applied the multiple regression analysis to determine regional gray matter density (rGMD) which is associated with the accuracy of object and spatial WM, respectively. Age, gender, and global density of GM were included as the confounding variables in the regression model. A threshold of corrected cluster *p* < 0.05 was set (voxel wise *p* < 0.001).

### Functional Connectivity Analysis

The resting-state fMRI data were preprocessed using the Data Processing Assistant for Resting-State fMRI (DPARSF^3^) incorporated in the MATLAB 2010a (Math Works, Natick, MA, United States) platform. The first 10 volumes of each functional image were discarded. The remaining 232 images were Slice Time Corrected and then realigned to the middle image volume to correct for head motion. Subsequently, all realigned images were spatially normalized to the standard template. The images were resampled into a 3 mm^3^ × 3 mm^3^ × 3 mm^3^ voxel, followed by spatial smoothing with a 6 mm FWHM. The smoothed images were linearly detrended, and we regressed out of global mean signal, white matter, cerebrospinal fluid, and 24 motion parameters for head movement. Finally, the images were filtered at 0.01–0.1 Hz.

Regions-based rsFC analysis were performed to calculate the FC maps of each seed ROI using the Resting–state fMRI DATA Analysis Toolkit (REST^4^), based on previous studies which showed that object WM is associated with the brain activity of the inferior frontal junction (IFJ) (Roth et al., 2006) and bilateral intraparietal sulcus (IPS) (Mecklinger et al., 2000). Spatial WM is associated with the brain activity of bilateral superior frontal sulcus (SFS) (Mohr et al., 2006a) and bilateral dlPFC (Yin et al., 2013). In addition, VBM analysis results showed the significant positive correlations between rGMD and the accuracy of object WM in the right middle occipital gyrus (MOG) and left superior temporal gyrus (STG), and the significant positive correlation between the right middle frontal gyrus (MFG) and the accuracy of spatial WM. We defined five ROIs for object WM according to previous rsFC studies and the VBM analysis results in the present study. These ROIs were defined as a sphere with a 6 mm radius centered at the left IFJ, bilateral IPS, left STG, and right MOG (Supplementary Table S1). Five ROIs were defined as a sphere with a 6 mm radius centered at the bilateral SFS, bilateral dlPFC, and the right MFG for spatial WM.

Then, the average time course of each ROI were extracted, and a correlation analysis was performed between the average time course of each ROI and the time course of each voxel in the whole brain, to obtain the FC map. The FC map was converted using Fisher’s r-to-z transformation to improve the normality. Finally, we applied multiple regressions to identify the brain regions where functional connectivity with ROIs were significantly related to the accuracy of object and spatial WM, respectively. Age and gender was included as covariates in the regression model. A threshold of corrected cluster *p* < 0.05 was set (voxel wise *p* < 0.001).

## Results

### Behavioral Data

Descriptive statistics for object and spatial WM and descriptive statistics of the demographic are reported in Table 1.

**TABLE 1:** Descriptive statistics of subject demographics and WM performance.

	**Mean**	***SD***	**Range**
Age	20.11	1.76	18–25
Object WM	0.787	0.081	0.516–0.950
Spatial WM	0.672	0.084	0.500–0.867

*WM, working memory.*

### VBM Analysis Result

**FIGURE 2:**
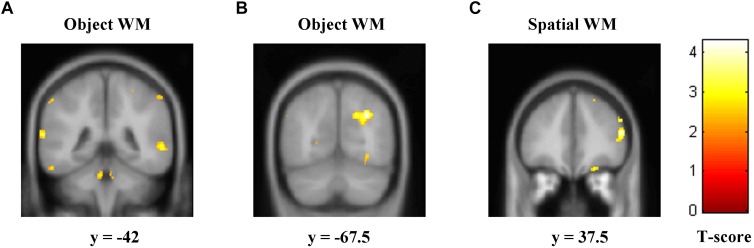
Regions of correlation between GMD and accuracy of object and spatial WM. The accuracy of object WM was positively correlated with GMD in two clusters that mainly contain **(A)** left STG, **(B)** right MOG, and **(C)**. The accuracy of spatial WM was positively correlated with GMD in the right MFG. GMD, gray matter density; WM, working memory; STG, superior temporal gyrus; MOG, middle occipital gyrus; MFG, middle frontal gyrus.

Correlation of rGMD with the accuracy of two types of WM with multiple regression analyses was used. We found that the left STG (Figure 2A and Table 2; cluster size = 383 voxels; peak coordinates in MNI: −66, −42, 13.5; *t*-peak = 3.99) and the right MOG (Figure 2B and Table 2; cluster size = 511 voxels; peak coordinates in MNI: 30, −67.5, 34.5; *t*-peak = 4.66) were positively correlated with the accuracy of object WM. We extracted the average GMD of the right MOG and left STG, and then correlated the average GMD with the accuracy of object WM. A significant positive correlation was observed between the average GMD of the right MOG and left STG and the accuracy of object WM (*r* = 0.62, *p* < 0.001^∗∗∗^). The accuracy of spatial WM was positively correlated with rGMD in the right MFG (Figure 2C and Table 2; cluster size = 408 voxels; peak coordinates in MNI: 51, 37.5, 15; *t*-peak = 4.36; *r* = 0.48, *p* < 0.001^∗∗∗^).

**TABLE 2:** Results of voxel-based morphometry analysis and functional connectivity analysis.

**VBM analysis result**		**Brain regions**	**Cluster size**	**Peak MNI**			**Peak *t*-value**
				***x***	***y***	***z***	
Object WM		Left STG	383	−66	−42	13.5	3.99
		Right MOG	511	30	−67.5	34.5	4.66
Spatial WM		Right MFG	408	51	37.5	15	4.36
**FC analysis result**							
Object WM	Seeds						
	Right IPS	Left PostCG	65	−48	−24	54	4.41
		Left SMA	128	−3	−18	60	4.47
		Right PreCG	60	39	−18	60	3.87
Spatial WM	Left dlPFC	Left precuneus	49	−6	−51	72	–5.16
	Right dlPFC	Right MFG	48	48	39	18	4.70
	Left SFS	Left SMA	134	−6	21	48	4.50
		Right IPL	62	45	−54	45	4.07

*MNI, Montreal Neurological Institute; STG, superior temporal gyrus; MOG, middle occipital gyrus; MFG, middle frontal gyrus; IPS, intraparietal sulcus; dlPFC, dorsolateral prefrontal cortex; SFS, superior frontal sulcus; SMA, Supplementary Motor Area; PreCG, precentral gyrus; PostCG, postcentral gyrus; IPL, inferior parietal lobe; WM, working memory.*

### Functional Connectivity Analysis Result

**FIGURE 3:**
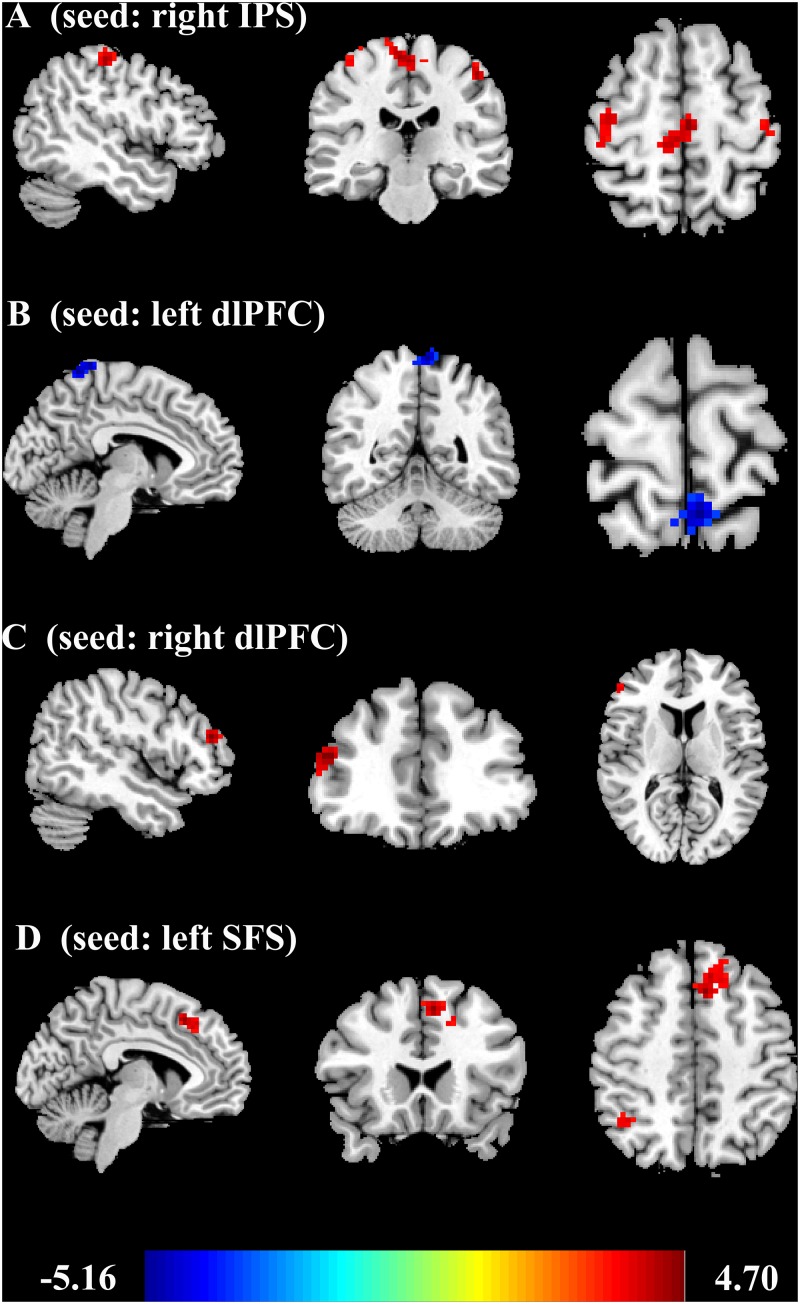
Correlations between ROIs and individual differences in object and spatial WM. **(A)** Region of a positive correlation between the strength of functional connectivity with right IPS and accuracy of object WM. **(B)** Region of a negative correlation between the strength of functional connectivity with left dlPFC and accuracy of spatial WM. **(C)** Region of a positive correlation between the strength of functional connectivity with right dlPFC and accuracy of spatial WM. **(D)** Region of a positive correlation between the strength of functional connectivity with left SFS and accuracy of spatial WM. These results are shown with a threshold of corrected cluster *p* < 0.05 (voxel wise *p* < 0.001) corrected for multiple comparisons using the AlphaSim program. IPS, intraparietal sulcus; dlPFC, dorsolateral prefrontal cortex; SFS, superior frontal sulcus; WM, working memory.

**FIGURE 4:**
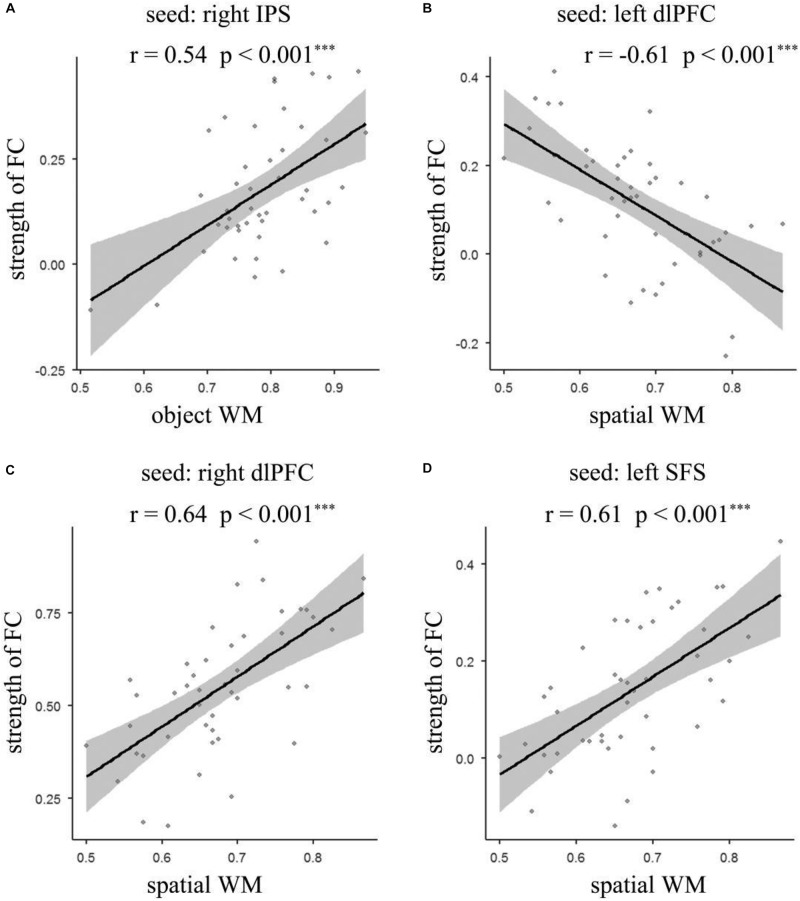
Scatter plot depicting significant correlations between the strength of functional connectivity and accuracy of object and spatial WM. **(A)** The correlation between the accuracy of object WM and functional connectivity between right IPS and the average strength of left PostCG, left SMA, and right PreCG; **(B)** the correlation between the accuracy of spatial WM and functional connectivity of left dlPFC – left Precuneus; **(C)** the correlation between the accuracy of spatial WM and functional connectivity of right dlPFC – right MFG; **(D)** the correlation between the accuracy of spatial WM and functional connectivity between left SFS and the average strength of left SMA and right IPL. IPS, intraparietal sulcus; PostCG, postcentral gyrus; PreCG, precentral gyrus; dlPFC, dorsolateral prefrontal cortex; MFG, middle frontal gyrus; SFS, superior frontal sulcus; SMA, Supplementary Motor Area; IPL, inferior parietal lobe; WM, working memory.

To identify the brain regions where the FC with pre-defined ROIs are significantly correlated with the accuracy of object and spatial WM, multiple linear regression analyses were performed separately. Age and gender were included as covariates in the regression model. The results (Figures 3A, 4A and Table 2) revealed that the accuracy of object WM was positively correlated with the strength of FC between the right IPS and left postcentral gyrus (PostCG), positively correlated with the strength of FC between the right IPS and left Supplementary Motor Area (SMA), and positively correlated with the strength of FC between the right IPS and right precentral gyrus (PreCG). The accuracy of spatial WM was significantly positively correlated with the strength of FC between the right dlPFC and right MFG (Figures 3C, 4C and Table 2), positively correlated with the strength of FC between the left SFS and right inferior parietal lobule (IPL) (Figures 3D, 4D and Table 2), and the strength of FC between left SFS and left SMA (Figure 3D and Table 2), as well as negatively correlated with the strength of FC between the left dlPFC and left precuneus (Figures 3B, 4B and Table 2). No significant results were found in other ROIs.

## Discussion

The present study aimed to investigate the associations between regional GMD as well as intrinsic functional connectivity underlying object and spatial WM. We found that the GMD of the right MOG and left STG were positively correlated with the accuracy of object WM. The accuracy of object WM is positively correlated with the FC strength of right IPS – left PostCG and the strength of right IPS – left SMA, as well as the strength of right IPS – right PreCG. The accuracy of spatial WM is positively correlated with the FC strength of right dlPFC – right MFG, the strength of left SFS – right IPL, and the strength of left SFS – left SMA, as well as negatively correlated with the strength of FC between the left dlPFC – left precuneus. Altogether, our findings extend previous studies by revealing the brain structures and the functional connectivity underlying object and spatial WM.

### Object Working Memory

The distribution of brain regions showed positive correlations with the accuracy of object WM in the right MOG and left STG. Previous findings showed that the performance of object WM was significantly correlated with the microstructure of the occipital-prefrontal fasciculus (Walsh et al., 2011). Song found that the occipital and the temporal cortices are responsible for processing the shape of an object (Song and Jiang, 2006). The lateral occipital cortex proved to be a crucial region for object recognition and representation (Sayres and Grillspector, 2008; Erdogan et al., 2016). Additionally, previous studies showed that the right MOG is associated with the detection of changes or exogenous attention in non-specific visual information processing (Tanaka et al., 2009). Our findings indicate that rGMD in the MOG is associated with a sensitivity to object change or gives more attention to the object and more quickly recognizes and represents the object. Furthermore, previous findings indicated that STG serves as a hub region where spatial information and object-based information are maintained (Park et al., 2011) and it is also associated with the detection of change in visual system (Tanaka et al., 2009), which may indicate that larger GMD in the STG may be associated with more sensitivity to object change and better maintains object information.

The strength of the FC between the right IPS and three regions (left PostCG, left SMA, and right PreCG) was significantly associated with the capacity of object WM. The right PreCG is the primary motor cortex controlling human behavior, and it is connected to the ventrolateral nucleus of the thalamus in order to cooperate in cognitive processing. The PostCG and PreCG was also activated in object recognition (Pollmann and von Cramon, 2000). The SMA was considered to be involved in a response preparatory set in VWM (Mecklinger et al., 2000; Pollmann and von Cramon, 2000). In the present study, the FC strength between the right IPS and left PostCG and right PreCG may be associated with the storage of object information, and the control for the behavioral response during the task or some cognition processing in WM. The FC strength between the right IPS and left SMA was associated with the movements of response.

### Spatial Working Memory

A significant positive correlation between rGMD and accuracy of spatial WM was found in the right MFG. The MFG lies in the dlPFC, as indicated by many previous studies (Sweeney et al., 1996; Carlson et al., 1998). The dlPFC plays an important role in monitoring and manipulating information in WM (Petrides et al., 2012). In several studies, lesions limited to the dlPFC impaired VWM (Goldman et al., 1971; Funahashi et al., 1993) and a delayed response tasks of primates showed that the dlPFC is associated more with the temporary maintenance of spatial information and its processing and is a critical region for spatial WM (Alekseichuk et al., 2016). Furthermore, the MFG is a key region of the Ventral Attention Network and is involved in bottom-top attention (Doricchi et al., 2010). It is also correlated with attentional control ability (Japee et al., 2015). Our findings suggest that larger GMD in the MFG might be associated with better maintaining of spatial information and more attention to the current task.

The significant positive associations between the accuracy of spatial WM and the strength of FC were identified in the right dlPFC – right MFG, left SFS – right IPL, and left SFS – left SMA. And a significant negative correlation between the accuracy of spatial WM and the strength of FC was found in the left dlPFC – left precuneus. Previous neuroimaging and patient studies have stressed the importance of dlPFC in monitoring and manipulating information in WM. The functional connectivity of right MFG was in line with our VBM findings, which again highlighted the importance of right MFG in spatial WM. In the present study, the FC strength between right dlPFC and right MFG was associated with the ability of keeping and manipulating the visual information. IPL was considered to play a key role in visual information integration. During ocular exploration, the involvement of IPL could contain maps of the whole visual field with underlying mechanisms of spatiotemporal maintenance of important information (Corbetta and Shulman, 2002). Activation in bilateral SFS was greater during spatial delays than during object delays (Joseph et al., 2003). Our findings of the FC strength between left SFS and right IPL may be involved in integrating and maintaining visual information. Altogether, our findings are in line with previous studies indicating that the ventral stream centered on dlPFC is crucial for the identification of objects (Leslie et al., 1998).

A comparison of the brain regions of the two tasks revealed differences between the ventral and dorsal pathways. The object WM recruited the ventral occipital – temporal regions, including the left STG, right MOG, and left PostCG. These brain regions are crucial for the identification of objects. In contrast, the spatial WM task recruited the dorsal frontal – parietal regions in the right MFG, bilateral dlPFC, and right IPL. These regions play a key role in the spatial perception and the visual guidance of movements toward objects in space. The functional connectivity of left SMA was found in both object and spatial WM, which may indicate that these two tasks require the involvement of response-preparatory processing. The SMA played a crucial role in preparing the actual response execution. These findings therefore provide evidence that processing these two tasks recruited different brain networks.

We would like to point out the limitation of the selection of stimulus. Operating on different types of stimulus characteristics is important for VWM. Previous studies have extensively studied these types of stimulus characteristics. Faces are widely used in object WM (Serences et al., 2004; Theeuwes and Stigchel, 2006; Joshua et al., 2007; Jiang et al., 2016), and color, shape, or direction of motion are extensively applied in spatial WM (Bichot et al., 2005; Heuer and Schubö, 2016). Previous research has paid little attention to the differences between these two types of tasks. It is possible that object and spatial WM recruit a largely overlapping neural network. On the basis of the present paradigm, we cannot rule out that only the brain regions related to object WM (or spatial WM) are activated in the object WM (or spatial WM) task. But our findings strongly support that these two types of WM activate different brain networks.

## Conclusion

In summary, the present study applied a morphometry analysis and rsFC to examine their association with the two subsystems of VWM. Our findings demonstrate that brain regions belonging to the ventral stream in both structure and functional connectivity were associated with object WM. In contrast, brain regions belonging to the dorsal stream were associated with spatial WM.

## Ethics Statement

This study was carried out in accordance with the recommendations of Institutional Review Board of the Southwest University Brain Imaging Center with written informed consent from all subjects. All subjects gave written informed consent in accordance with the Declaration of Helsinki. The protocol was approved by the Institutional Review Board of the Southwest University Brain Imaging Center.

## Author Contributions

ZR and YZ contributed to the analyzing data and completing the manuscript. ZR took part in reanalyzing data, responding to reviewers, and improving the English for the whole manuscript. YZ contributed to analyzing data and completing the manuscript draft. HH and QF proposed the idea of the study. TB and JQ provided technical support for carrying out the experiments. All of the above authors contributed to data acquisition and conducted the experiment. We also extend our gratitude to all participants.

## Conflict of Interest Statement

The authors declare that the research was conducted in the absence of any commercial or financial relationships that could be construed as a potential conflict of interest.

## Footnotes

1 http://www.mathworks.com

2 www.fil.ion.ucl.ac.uk/spm

3 http://resting-fmri.sourceforge.net

4 http://www.restfmri.net
