# Supplementary material for: The Different Brain Mechanisms of Object and Spatial Working Memory: Voxel-Based Morphometry and Resting-State Functional Connectivity
Source: Front Hum Neurosci. 2019 Jul 19;13:248. doi: 10.3389/fnhum.2019.00248 (PMC6659551; doi:10.3389/fnhum.2019.00248)
Supplement: Supplementary file 1 [file Table_1.DOC]

Table S1 The Montreal Neurological Institute (MNI) coordinates of the pre-defined ROIs

| ROIs | MNI coordinates | | | References |
| --- | --- | --- | --- | --- |
|  | X | Y | Z |  |
| **Object WM**  Left STG | -66 | -42 | 13.5 | VBM analysis result |
| Right MOG | 30 | -67.5 | 34.5 | VBM analysis result |
| Left IPS | -37 | -58 | 37 |  |
| Rihgt IPS | 36 | -50 | 45 |  |
| Left IFJ | -39.8 | -23.9 | 27.1 |  |
|  |  |  |  |  |
| **Spatial WM**  Right MFG | 51 | 37.5 | 15 | VBM analysis results |
| Left dlPFC | -37 | 23 | 27 |  |
| Right dfPFC | 41 | 35 | 29 |  |
| Left SFS | -24 | -8 | 61 |  |
| Right SFS | 28 | -6 | 55 |  |

References

A. Mecklinger, V. B., C. Gruenewald, S. Bentin, D.Y. von Cramon. (2000). What Have Klingon Letters and Faces in Common? An fMRI Study on Content-Specific Working Memory Systems. *Human Brain Mapping, 11*, 146-161.

Mohr, H. M., Goebel, R., & Linden, D. E. (2006). Content- and task-specific dissociations of frontal activity during maintenance and manipulation in visual working memory. *J Neurosci, 26*(17), 4465-4471.

Roth, J. K., Serences, J. T., & Courtney, S. M. (2006). Neural system for controlling the contents of object working memory in humans. *Cereb Cortex, 16*(11), 1595-1603.

Yin, J.-J., Liao, L.-M., Luo, D.-X., Xu, K., Ma, S.-H., Wang, Z.-X., . . . Zhang, J. (2013). Spatial Working Memory Impairment in Subclinical Hypothyroidism: An fMRI Study. *Neuroendocrinology, 97*(3), 260-270.
